# Comprehensive whole-genome characterization of SARS-CoV-2 strains in Jining China 2024–2025

**DOI:** 10.3389/fmicb.2026.1798666

**Published:** 2026-05-08

**Authors:** Haixia Yang, Xiaoyu Wang, Linlin Zhang, Lingming Kong, Wei Liu, Qiang Yin, Yongjian Jia, Huixin Dou, Ting Chen, Feifei He, Lili Zhang, Tihui Wang, Yajuan Jiang, Boyan Jiao

**Affiliations:** 1 College of Medical Imaging and Laboratory Medicine, Jining Medical University, Jining, China; 2 Department of Laboratory, Jining Center for Disease Control and Prevention, Jining, China; 3 Jining Key Laboratory of Infectious Disease Control and Prevention, Jining, China; 4 Department of Laboratory, Yanzhou District Center for Disease Control and Prevention, Jining, Shandong, China; 5 Department of AI and Bioinformatics, Nanjing Chengshi BioTech (TheraRNA) Co. Ltd., Nanjing, China; 6 Jining Center for Disease Control and Prevention, Jining, China; 7 School of Basic Medicine, Jining Medical University, Jining, China; 8 Computer Information Technology, Northern Arizona University, Flagstaff, AZ, United States; 9 Department for Chronic Non-communicable Disease Prevention and Control, Jining Center for Disease Control and Prevention, Jining, China

**Keywords:** epidemiology, open reading frame 8, severe acute respiratory syndrome coronavirus 2, spike protein, whole genome sequencing

## Abstract

**Objective:**

To characterize the whole-genome features and evolutionary variation patterns of severe acute respiratory syndrome coronavirus 2 circulating in Jining City during 2024–2025.

**Methods:**

Whole-genome sequencing was performed on clinical specimens collected from SARS-CoV-2–infected cases in Jining City during 2024–2025 using next-generation sequencing technology. Phylogenetic and mutation analyses were conducted using standard bioinformatics tools.

**Results:**

A total of 429 complete SARS-CoV-2 genome sequences were obtained between January 2024 and December 2025, including 240 sequences in 2024 and 189 sequences in 2025. In 2024, circulating strains showed a sequential replacement from the XBB.1.9 lineage to the BA.2.86 lineage and subsequently to the XDV.1 lineage. All sequences obtained in 2025 belonged to the XDV.1 lineage and its sublineages. Whole-genome nucleotide similarity between the 429 sequences and the Wuhan-Hu-1 reference strain ranged from 99.64 to 99.81%, with the lowest nucleotide similarity observed in the ORF6 and spike genes. Amino acid mutation analysis revealed that the spike proteins of the XBB.1.9, BA.2.86, and XDV.1 lineages harbored an average of 42.67, 53.61, and 55.15 amino acid mutation, respectively. The XDV.1 lineage and its sublineages showed the greatest number of spike protein amino acid substitutions. In addition, ORF8 G8 stop and ORF8 Q18 stop mutations were identified, and frameshift mutations in ORF8 were detected in 10 sequences.

**Conclusion:**

Different Omicron lineages circulated alternately in Jining City during 2024–2025, with XDV.1 and its sublineages becoming the predominant circulating strains in 2025. These findings highlight the critical importance of continuous genomic surveillance for real-time monitoring of viral evolution, and provide key data to support the timely adjustment of vaccines and public health strategies in response to emerging variants. Continuous whole-genome sequencing and analysis of SARS-CoV-2 are essential for timely monitoring of viral evolution and provide a theoretical basis for effective prevention and control strategies.

## 1 Introduction

Since its emergence in 2019, severe acute respiratory syndrome coronavirus 2 has caused large-scale outbreaks worldwide, posing a serious threat to global public health security and human life (Platto et al., 2020; Yuan et al., 2023; Yin et al., 2024). SARS-CoV-2 continues to evolve, giving rise to multiple variants of concern, including Alpha, Beta, Gamma, Delta, and Omicron. Following its initial detection in November 2021, the Omicron variant rapidly became the dominant global variant due to its enhanced transmissibility and immune evasion capacity (Callaway, 2021; Ghosh et al., 2022; Viana et al., 2022). The ongoing evolution of the Omicron variant has led to the emergence of numerous sublineages, such as XBB, BA.2.86, and XDV (You et al., 2025; Yan et al., 2025; Yuan et al., 2025). These distinct lineages differ in genomic characteristics, transmissibility, and immune escape potential, presenting substantial challenges for the prevention and control of SARS-CoV-2 worldwide (Liu et al., 2024).

SARS-CoV-2 is a single-stranded positive-sense RNA virus with a genome of approximately 30 kb, encoding four structural proteins—nucleocapsid protein, matrix protein, envelope protein, and spike protein—as well as multiple nonstructural proteins (Yuan et al., 2025). The spike protein mediates host receptor recognition and induces neutralizing antibody responses. Owing to its high mutation rate, amino acid substitutions in the spike protein represent a major mechanism by which the virus adapts to host immune pressure. Such mutations can enhance binding affinity to host receptors and reduce the protective efficacy of immunity induced by vaccination or prior infection (Wang et al., 2025; Yamamoto and Noguchi, 2025; Argyrou et al., 2025). Several types of SARS-CoV-2 vaccines, including mRNA vaccines, inactivated vaccines, and protein subunit vaccines, have been developed worldwide. Despite their distinct technological approaches, the majority of these vaccines target the spike protein of SARS-CoV-2. In addition, the ORF8 protein has been shown to be closely associated with viral pathogenicity and immune evasion (Valcarcel et al., 2021; Vinjamuri et al., 2022). Multiple types of premature termination mutations have been identified in ORF8. The multiple premature termination mutations identified in ORF8 disrupt its key functional domains, potentially attenuating ORF8-mediated virulence, which may facilitate higher host survival rates and enhance transmission opportunities (Yin et al., 2024; Vinjamuri et al., 2022; Pereira, 2021).

Jining City has a population of approximately 8.19 million, which provides potential conditions for local transmission of SARS-CoV-2. The Beijing-Hangzhou Grand Canal traverses the city, connecting it to the Yangtze River and providing direct access to the sea, which improves the efficiency of cross-regional population movement, creating favorable conditions for the importation and spread of SARS-CoV-2. Moreover, as the cradle of Confucian culture and a world-renowned tourist destination, Jining attracts approximately 100 million visitors annually, including over 100,000 international tourists, which increases the risk of viral importation from overseas and other domestic regions. Consequently, Jining serves as a representative area for studying the prevalence and genomic evolution of SARS-CoV-2. In this study, whole-genome sequencing and bioinformatic analyses were performed on SARS-CoV-2 strains collected in Jining City during 2024–2025. The aim was to characterize the genomic features and evolutionary patterns of circulating SARS-CoV-2 strains, to identify key mutations in the spike protein, and to describe termination and frameshift mutations in ORF8. These findings provide a scientific basis for risk assessment and inform strategies for the prevention and control of SARS-CoV-2.

## 2 Materials and methods

### 2.1 Sample collection

Nasopharyngeal swab specimens were collected from 429 laboratory-confirmed SARS-CoV-2–infected individuals diagnosed at 11 county-level general medical institutions, Jining No.1 People’s Hospital, and The Affiliated Hospital of Jining Medical University in Jining City between January 2024 and December 2025.

### 2.2 Whole-genome sequencing of SARS-CoV-2

Viral nucleic acids were extracted from 200 μL of nasopharyngeal swab specimens using the GeneRotex 96 automated nucleic acid extraction system (Xi’an Tianlong Science and Technology Co., Ltd.) with the qEx-DNA/RNA Viral Nucleic Acid Extraction Kit (Cat. No. T183). SARS-CoV-2 nucleic acid detection was performed using a fluorescent PCR-based SARS-CoV-2 detection kit (Shanghai Berger Medical Technology Co., Ltd.; Cat. No. ZC-HX-201-2). Positive samples with cycle threshold (Ct) values ≤32 were selected for whole-genome sequencing.

Library preparation and whole-genome capture were conducted using the VAHTS RNA Multi-PCR Library Prep Kit (Cat. No. NA-211-C4) and VAHTS DNA Clean Beads (Cat. No. N411-01-AA) (Nanjing Vazyme Biotech Co., Ltd.). Nucleic acid concentrations were quantified using a Qubit 3 Fluorometer (Invitrogen). Sequencing was performed on an Illumina MiSeq platform using the MiSeq Reagent Micro Kit v2 (300 cycles; Cat. No. 15036715).

### 2.3 Bioinformatic analysis

Raw sequencing data were assembled using CLC Genomics Workbench version 25 (QIAGEN) with the Wuhan-Hu-1 reference genome (GenBank accession number MN908947.3). The low-coverage definition threshold was set to 30. Lineage assignment and mutation analysis were performed using Pangolin v4.3 (https://pangolin.cog-uk.io/) and Nextclade v3.18.1 (https://clades.nextstrain.org/).

The early SARS-CoV-2 isolate Wuhan-Hu-1 sequence was downloaded from the NCBI database. Multiple sequence alignment was conducted using MAFFT v7.487 (Katoh and Standley, 2013). Phylogenetic trees were constructed using IQ-TREE v2.1.4 based on the maximum-likelihood method with 1,000 bootstrap replicates (Nguyen et al., 2015). Tree visualization and annotation were performed using FigTree v1.4.2 and iTOL (Letunic and Bork, 2024). Genome sequence similarity analysis was carried out using MEGA v7.0.14. The tertiary structure of the ORF8 protein was predicted using HelixFold3.

In the Search section of the GISAID EpiCoV database, select January 1, 2024 to December 31, 2025 in the Collection field, and enter G27915T, C27945T, or del28254 in the Nucl Mutations field to obtain the number of SARS-CoV-2 sequences and the number of SARS-CoV-2 sequences carrying the G27915T, C27945T, or A28254 deletion mutations collected between January 1, 2024 and December 31, 2025.

### 2.4 Statistical analysis

All statistical analyses were conducted using SPSS version 25.0. The chi-square (χ^2^) test was employed to compare the proportions of the G27915T, C27945T, and A28254del variants. One-way ANOVA followed by post-hoc analysis was used to compare sequence similarity across different SARS-CoV-2 genes and the number of spike protein amino acid mutations among different sublineages. A *p*-value < 0.05 was considered statistically significant.

## 3 Results

### 3.1 Genomic characteristics of SARS-CoV-2

Between January 2024 and December 2025, a total of 5,776 SARS-CoV-2 positive infections were detected, and a total of 429 SARS-CoV-2 whole-genome sequences with genome coverage exceeding 98% were obtained in Jining City (Supplementary Table 1). 429 sequences were deposited in the GISAID database. Detailed metadata, including collection date, patient demographics (age and sex), clinical classification, sequencing coverage, and GISAID IDs, are provided in Supplementary Table 2. Among these, 240 sequences were generated in 2024 and were designated as hCoV-19/China/Shandong-Jining-001/2024 to hCoV-19/China/Shandong-Jining-240/2024. In 2025, 189 sequences were obtained and designated as hCoV-19/China/Shandong-Jining-001/2025 to hCoV-19/China/Shandong-Jining-189/2025.

**Figure 1:**
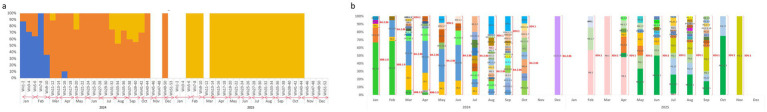
Temporal distribution of SARS-CoV-2 variant genotypes detected in Jining City during 2024–2025. Sequence analysis was performed using Nextclade v3.18.1. **(A)** According to the temporal distribution of the SARS-CoV-2 XBB.1.9 lineage, BA.2.86 lineage, and XDV.1 lineage. **(B)** According to the temporal distribution of SARS-CoV-2 sublineages. From November 2024 to January 2025, SARS-CoV-2 prevalence persisted at low levels in China (https://www.chinacdc.cn/jksj/xgbdyq/202502/t20250213_304257.html), resulting in a scarcity of samples available for sequencing. No SARS-CoV-2 whole-genome sequences were obtained in November 2024, January 2025, or December 2025.

Lineage assignment of the 429 SARS-CoV-2 genomes was performed using Nextclade v3.18.1 (Supplementary Figure 1). All sequences belonged to the Omicron variant. Among the 240 sequences obtained in 2024, three major lineages were identified. The most predominant XBB.1.9 sublineage was HK.3.2, with 30 sequences. The most predominant BA.2.86 sublineage was JN.1.16, with 43 sequences. The most predominant XDV.1 sublineage was XDV.1, with 17 sequences (Figure 1; Supplementary Figure 2; Supplementary Table 3). All 189 sequences obtained in 2025 belonged to the XDV.1 lineage and its sublineages. The most predominant XDV.1 sublineage was NB.1.8.1, with 48 sequences (Figure 1; Supplementary Figure 3; Supplementary Table 3). Temporal analysis revealed that the XBB.1.9 lineage was the predominant circulating lineage in Jining City from January to February 2024. From March to June 2024, BA.2.86 became the dominant lineage. Between July and December 2024, BA.2.86 and XDV.1 co-circulated. The XDV.1 lineage was the predominant circulating lineage in 2025 (Figure 1; Supplementary Figure 2; Supplementary Table 3).

The detailed distribution of the 429 SARS-CoV-2 sequences by lineage across the 11 counties in Jining City during 2024–2025 is presented in Supplementary Figure 4.

### 3.2 Phylogenetic analysis

Early SARS-CoV-2 isolate sequences, Alpha, Beta, Gamma, Delta, Omicron BA.1, BA.2, BA.2.75, BA.2.86, BA.3, BA.4, and BA.5 variant sequences were retrieved from the GISAID database. These reference sequences, together with 429 whole-genome SARS-CoV-2 sequences generated in this study and the Wuhan reference strain Wuhan-Hu-1/2019, were used to construct phylogenetic trees. The phylogenetic clustering results were consistent with lineage assignments obtained using Nextclade v3.18.1.

**Figure 2:**
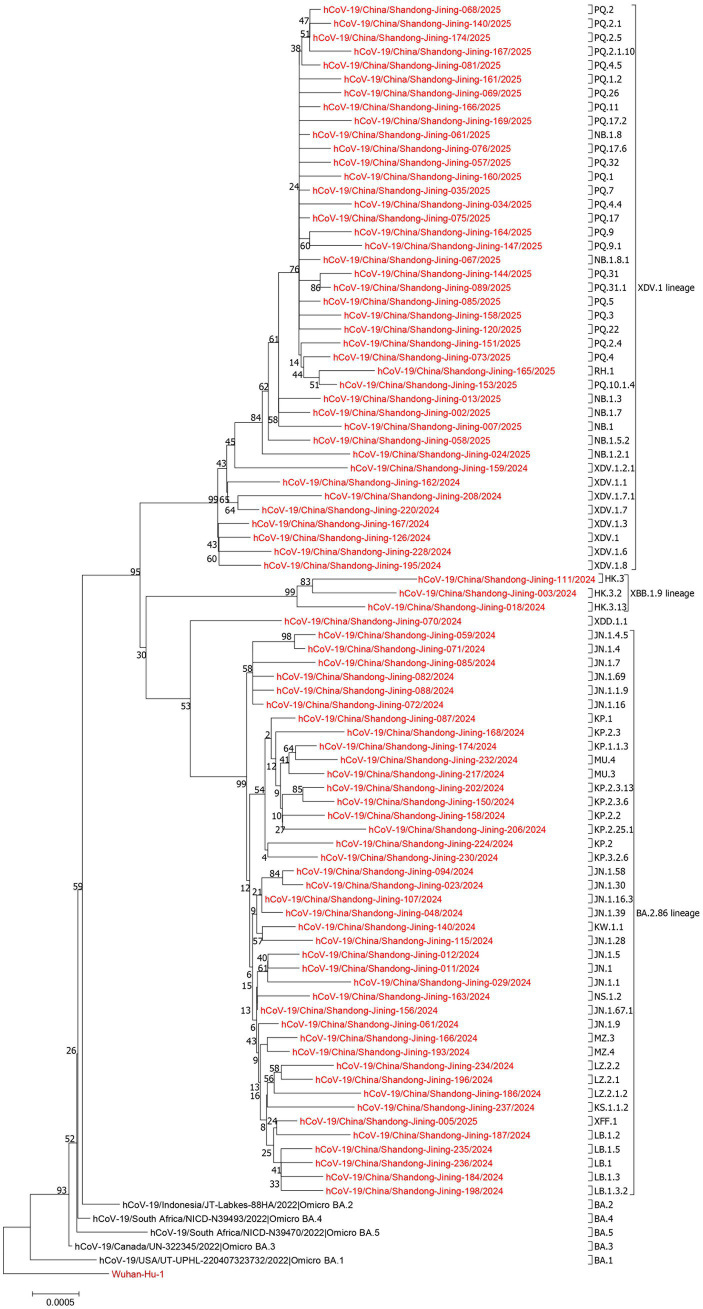
Phylogenetic analysis of 429 SARS-CoV-2 whole-genome sequences circulating in Jining City during 2024–2025. Multiple sequence alignment was performed using MAFFT v7.487. Phylogenetic trees were constructed using IQ-TREE v2.1.4 based on the maximum-likelihood method with 1,000 bootstrap replicates. Tree visualization and annotation were performed using FigTree v1.4.2 and iTOL. Red indicates SARS-CoV-2 sequences in Jining City. Red indicates sequences from Jining City, while brown represents the Wuhan-Hu-1 reference sequence.

In 2024, a total of 240 sequences predominantly clustered into the XBB.1.9, BA.2.86, and XDV.1 lineages. In 2025, 189 sequences were mainly grouped within the XDV.1 lineage (Figure 2).

### 3.3 Sequence similarity analysis

**Figure 3:**
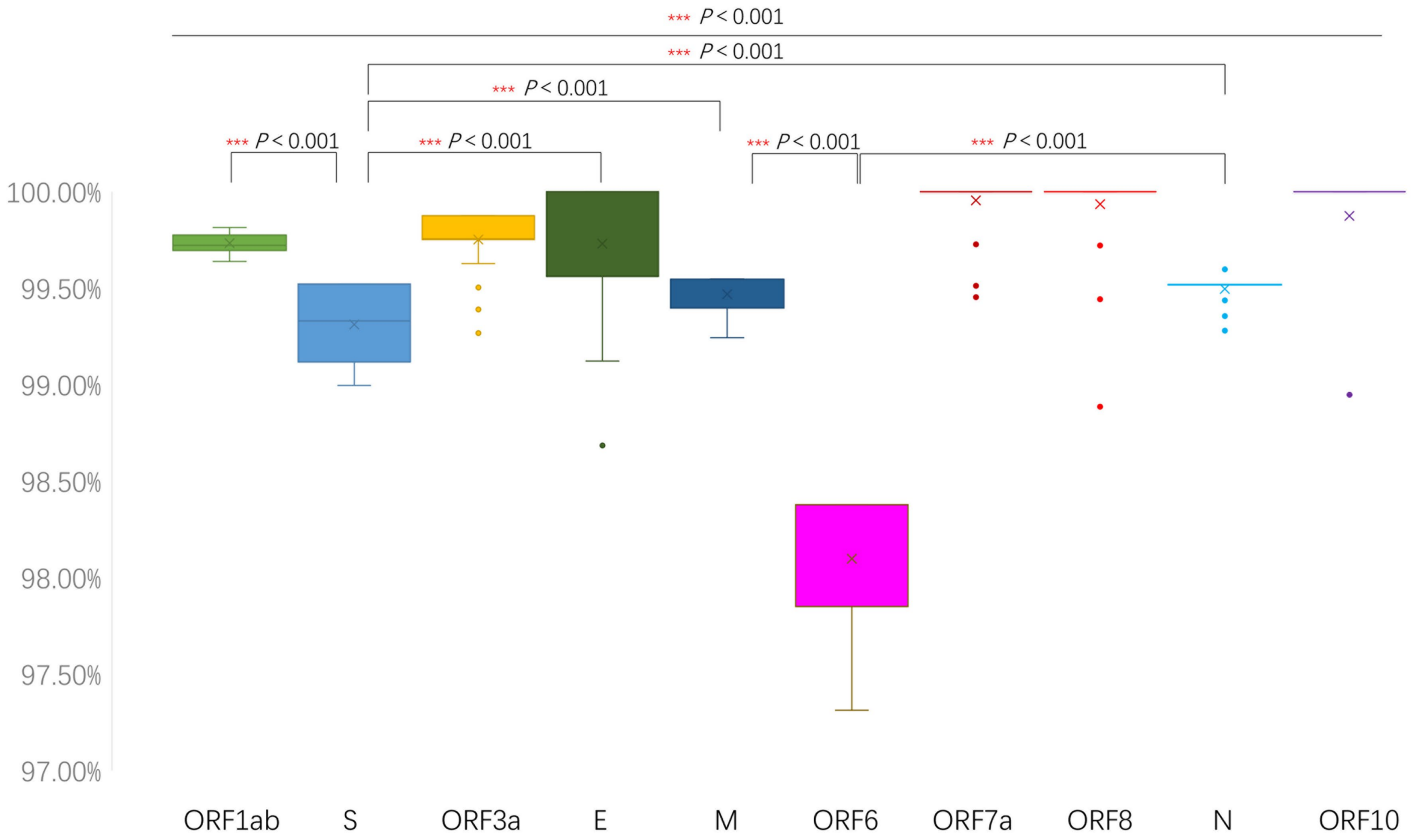
Sequence similarity analysis of 429 SARS-CoV-2 genomes obtained in Jining City during 2024–2025. Genome-wide similarity between the 429 sequences and the Wuhan-Hu-1 reference strain. The similarity values (in percentage) indicate the degree of sequence identity compared to the Wuhan-Hu-1 reference strain.

Using Wuhan-Hu-1 as the reference sequence, whole-genome nucleotide similarity between the 429 SARS-CoV-2 genomes obtained in Jining City during 2024–2025 and the Wuhan-Hu-1 strain ranged from 99.64 to 99.81%. Among all genomic regions, the ORF6 and spike genes exhibited the lowest nucleotide similarity (*p <* 0.001), with similarity ranges of 97.31–98.38% and 99.00–99.52%, respectively (Figure 3). The results indicated that the ORF6 and S genes were the most variable regions of SARS-CoV-2. The S gene encodes the spike protein, and the continuous accumulation of amino acid mutations in the spike protein is a core driver of the virus’s enhanced immune evasion and transmissibility.

The nucleotide similarity between representative sublineages—including HK.3.2, JN.1, JN.1.4.5, JN.1.16, JN.1.67.1, NB.1, NB.1.8.1, PQ.1, PQ.2, and PQ.17—and the Wuhan-Hu-1 reference strain is shown in Supplementary Table 4. No hypervariability specific to any single sublineage was observed, indicating a convergent pattern of variation among strains of different sublineages and reflecting a consistent evolutionary direction across them.

### 3.4 Spike protein mutation analysis

Compared with the Wuhan-Hu-1 reference sequence, the spike protein of the 429 SARS-CoV-2 genomes obtained in Jining City during 2024–2025 harbored 42–59 amino acid substitutions. In Jining City, the spike proteins of the XBB.1.9, BA.2.86, and XDV.1 lineages harbored an average of 42.67, 53.61, and 55.15 amino acid mutation, respectively. A total of 27 shared mutation sites were identified across all sequences, which were mainly located in the N-terminal domain (NTD) and the receptor-binding domain (RBD) (Supplementary Figure 5).

Compared with the HK.3.2 sublineage, the JN.1 sublineage exhibited amino acid differences in the NTD, RBD, RBM, CTD1, CTD2, and HR1 of the spike protein. Compared with the JN.1 sublineage, the XDV.1 lineage exhibited amino acid differences in the NTD and RBD of the spike protein. Furthermore, amino acid variations in the spike protein were also observed among XDV.1 lineages, including NB.1, NB.1.8.1, PQ.1, PQ.2, and PQ.17 (Supplementary Figure 5).

**Figure 4:**
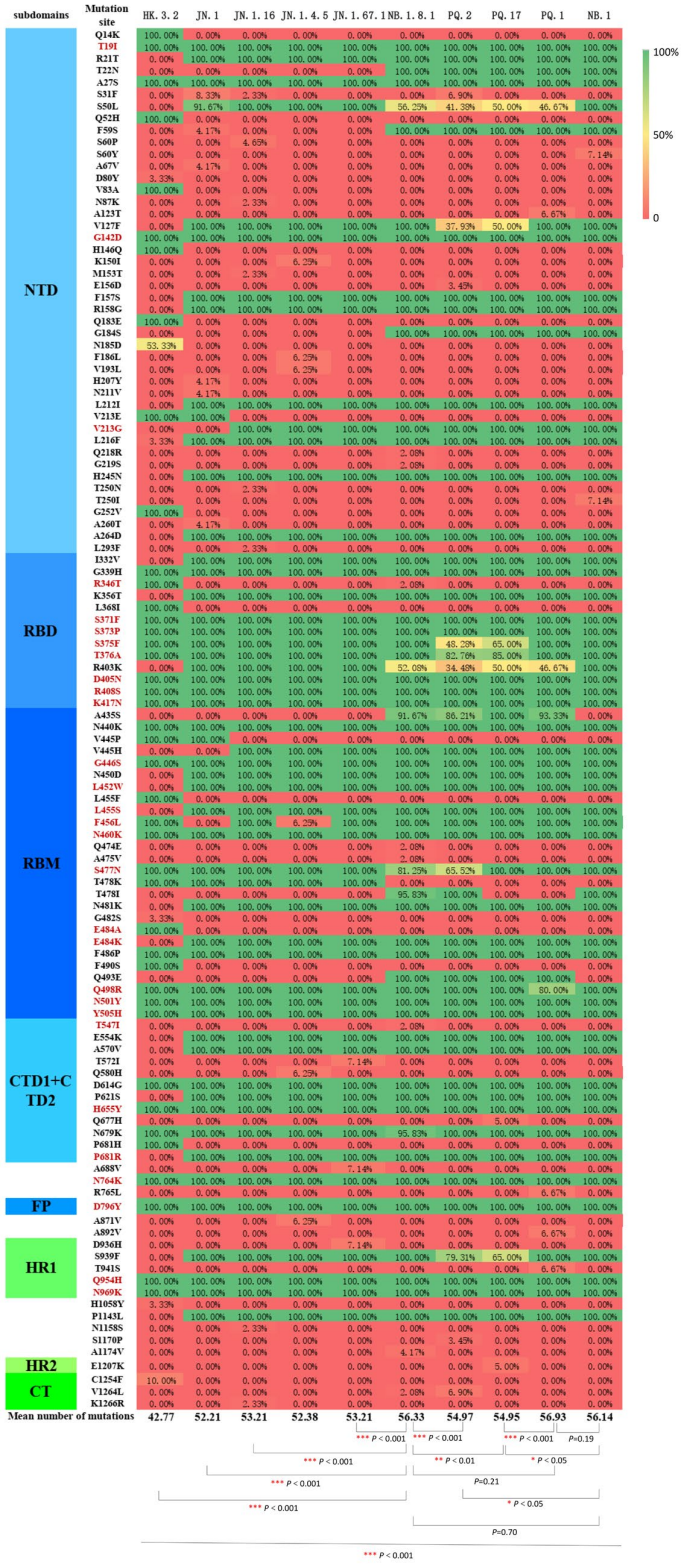
Clustering heatmap of spike protein amino acid mutation frequencies among SARS-CoV-2 sequences circulating in Jining City during 2024–2025. Spike protein mutation sites were identified using Nextclade v3.18.1. Rows represent spike protein amino acid mutation sites, and columns represent sublineages. The color scale represents the proportion of mutations. Red indicates low mutation frequency, yellow indicates medium mutation frequency, whereas green indicates high mutation frequency. Mutation sites marked in coffee brown represent known escape mutations.

Clustering heatmap analysis of spike protein amino acid mutation sites across different genotypes indicated that the NB.1.8.1, PQ.1, NB.1 sublineages exhibited the greatest number of spike protein amino acid mutations (*p* < 0.001) (Figure 4).

### 3.5 Analysis of SARS-CoV-2 ORF8 termination and frameshift mutations

**Figure 5:**
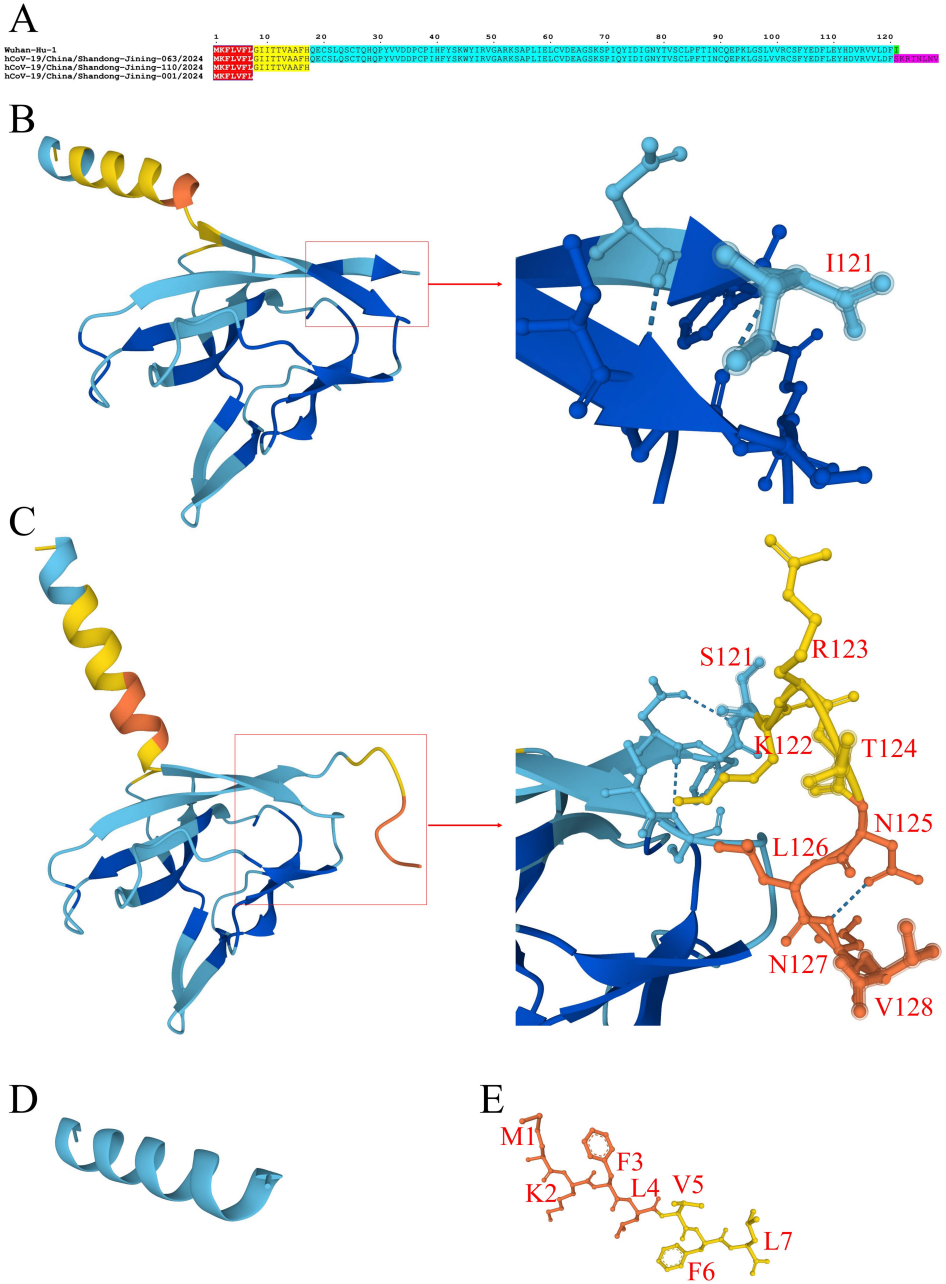
Analysis of ORF8 termination and frameshift mutations in SARS-CoV-2. Sequence alignment was performed using the ESPript 3.2 online tool. Tertiary structures of ORF8 proteins were predicted using the HelixFold3 online platform. **(A)** Amino acid sequence analysis of ORF8 termination and frameshift mutations. The hCoV-19/China/Shandong-Jining-063/2024 strain encodes an ORF8 frameshift mutant protein; hCoV-19/China/Shandong-Jining-110/2024 encodes an ORF8 Q18 stop protein; and hCoV-19/China/Shandong-Jining-001/2024 encodes an ORF8 G8 stop protein. **(B)** Tertiary structure of the ORF8 protein encoded by the Wuhan-Hu-1 reference strain. **(C)** Tertiary structure of the ORF8 frameshift mutant protein encoded by hCoV-19/China/Shandong-Jining-063/2024. **(D)** Tertiary structure of the ORF8 Q18 stop protein encoded by hCoV-19/China/Shandong-Jining-110/2024. **(E)** Tertiary structure of the ORF8 G8 stop protein encoded by hCoV-19/China/Shandong-Jining-001/2024. **(B–E)** 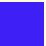 indicates prediction confidence based on pLDDT scores: very high confidence (pLDDT>90), 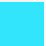 indicates prediction confidence based on pLDDT scores: very high confidence (pLDDT>90), 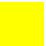 low confidence (50 < pLDDT≤70), 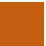 very low confidence (pLDDT ≤ 50).

The ORF8 gene is prone to multiple types of stop mutations. Among the 429 SARS-CoV-2 genomes analyzed, a G27915T substitution was identified in 30 HK.3.2 sublineage sequences, four HK.3.13 sublineage sequences, one HK.3 sublineage sequence, and one XDD.1.1 sublineage sequence. This mutation converted the codon encoding the eighth amino acid of the ORF8 protein from GGA to the stop codon TGA, resulting in premature termination at position 8 and the generation of a truncated ORF8 G8 stop protein (Figures 5A,B,E).

In addition, a C27945T substitution was detected in one JN.1 sublineage sequence and one JN.1.67.1 sublineage sequence. This mutation changed the codon encoding the eighteenth amino acid of the ORF8 protein from CAA to the stop codon TAA, leading to premature termination at position 18 and the formation of a truncated ORF8 Q18 stop protein (Figures 5A−C).

The generation of the ORF8 G8 stop protein and ORF8 Q18 stop protein leads to the loss of the amino acid sequence at positions 62–85 responsible for Interferon Regulatory Factor 3 (IRF3) binding, as well as the amino acid residues S24, V62, and L84 critical for Interleukin-17 Receptor A (IL-17RA) binding in the ORF8 protein. Consequently, these truncated proteins lose the domain to bind IRF3 and IL-17RA, thereby might impair the immunomodulatory function mediated by the ORF8 protein.

Furthermore, an A28254 deletion was identified in one HK.3.2 sublineage sequence (also carrying the G8 stop mutation), four HK.3.13 sublineage sequences (also carrying the G8 stop mutation), one JN.1 sublineage sequence, one JN.1.16 sublineage sequence, one XDV.1 sublineage sequence, one PQ.2 sublineage sequence, and one PQ.9.1 sublineage sequence. This deletion caused a frameshift mutation in ORF8, resulting in a shift of the C-terminal amino acid at position I121 to SKRTNLNV and the addition of a terminal peptide segment at the C-terminus of the ORF8 protein (Figures 5A,B,C). The frameshift and C-terminal extension may alter the spatial conformation of ORF8 and enable ORF8 to gain novel and potential protein–protein interaction interfaces, thereby affecting its interaction with host molecules.

The frequencies of the G27915T, C27945T, and A28254 deletion variants in Jining City during 2024–2025 were 8.39% (36/429), 0.47% (2/429), and 2.33% (10/429), respectively. Based on the GISAID database, as of March 11, 2026, the global frequencies of the G27915T, C27945T, and A28254 deletion variants among SARS-CoV-2 sequences submitted during 2024–2025 were 2.96% (23,481/793,099), 0.18% (1,426/793,099), and 1.60% (12,665/793,099), respectively. According to GISAID database, the G27915T mutation was first identified in March 14, 2020 and is one of the characteristic mutations of the XBB.1 lineage. The frequency of the G27915T variant among XBB.1 lineage was 95.32% (974,854/1,022,677). In contrast, The C27945T and A28254 deletion variants did not become characteristic mutations of the lineage of SARS-CoV-2, and no statistically significant differences were observed for the C27945T and A28254 deletion variants (χ^2^ = 1.96, *p* = 0.16; χ^2^ = 1.47, *p* = 0.23), indicating the unique evolutionary features of the local SARS-CoV-2 strains. Notably, the global sequence distribution within the GISAID database may be uneven and subject to geographical bias, which could potentially compromise the comprehensiveness and objectivity of comparative analyses between local sequences from Jining City and global sequences.

## 4 Discussion

Viral infections remain a major threat to human health, causing a wide spectrum of diseases ranging from acute infections to chronic conditions, long-term sequelae, and death, and continue to pose substantial challenges to global public health (Duan et al., 2018; Li et al., 2025; Rabe et al., 2025; Liu et al., 2025). Jining City is located at a key geographic crossroads in southwestern Shandong Province, with frequent population movement and interregional connectivity, which is likely to facilitate the introduction, spread, and local circulation of viral pathogens from surrounding regions (Yin et al., 2024; Dou et al., 2025; Fu et al., 2025; Jiang et al., 2024; Wu et al., 2024; Zhao et al., 2025). A total of 429 whole-genome SARS-CoV-2 sequences were obtained from Jining City during 2024–2025. The results showed that SARS-CoV-2 variants in Jining City circulated sequentially in the order of the XBB.1.9 lineage, followed by the BA.2.86 lineage and subsequently the XDV.1 lineage, which was generally consistent with nationwide trends in China during the same period (https://www.chinacdc.cn/jksj/xgbdyq/202601/t20260109_314543.html). However, notable regional differences were observed. At the national level, the XDV lineage was first detected as early as February 2024, and its proportion exceeded 40% by July 2024 (https://www.chinacdc.cn/jksj/xgbdyq/202408/t20240827_295731.html). In contrast, XDV was first detected in Jining City in May 2024, and its proportion did not reach 39.47% until August 2024, indicating a delayed emergence and spread compared with the national trend. However, a limitation of this study is that sequencing was restricted to samples with Ct values ≤ 32, excluding samples with low viral loads, which may have impacted the accuracy of our lineage frequency estimates.

Regional heterogeneity in the timing of XDV emergence has also been reported across China. For example, XDV was detected in Chongqing, located in central China, in April 2024 (Viana et al., 2022), whereas a study from Henan Province in northern China reported the first detection of XDV in July 2024 (Yuan et al., 2025). These spatial differences suggest that XDV may have spread gradually across China through population movement. In 2025, the predominant circulating lineage in Jining City was XDV.1, consistent with nationwide observations. These results underscore the continuous potential for the emergence of novel SARS-CoV-2 lineages. Consequently, it is imperative to bolster genomic surveillance and to promptly evaluate vaccine protective efficacy whenever a new lineage achieves widespread circulation. They also underscore the importance of enhancing surveillance and early warning systems to monitor the ongoing evolution and transmission dynamics of new lineage. Notably, this study lacks data on the travel history of the study subjects, making it difficult to accurately determine whether the SARS-CoV-2 lineages originated from local evolution or were introduced by domestic migrant populations or overseas travelers. This limitation may affect the precise interpretation of the epidemiological characteristics.

Similarity analysis indicated that the ORF6 and S genes are susceptible to mutation. As an interferon antagonist, the high variability of ORF6 may potentially alter the viral immune evasion capacity (Hoffmann et al., 2020; Mannar et al., 2022; Abduljaleel, 2025). The spike protein is the key viral protein mediating receptor binding and host immune recognition. Previous studies have shown that the Omicron variant harbors extensive amino acid substitutions in the spike protein, which directly affect viral transmissibility, pathogenicity, and immune escape potential (Hoffmann et al., 2020; Mannar et al., 2022; Abduljaleel, 2025). Spike protein mutations are predominantly concentrated in the N-terminal domain (NTD) and the receptor-binding domain (RBD), both of which are directly involved in host receptor engagement. Amino acid substitutions in these regions can substantially influence viral transmissibility (Ataya et al., 2025). In the present study, the spike gene exhibited relatively lower nucleotide similarity to the Wuhan-Hu-1 reference strain, and 43–59 amino acid substitutions were identified in the spike protein of SARS-CoV-2 strains circulating in Jining City during 2024–2025. These substitutions were mainly located in the NTD and RBD, further indicating continuous adaptive evolution of SARS-CoV-2 under immune pressure.

Notably, the number of spike protein amino acid substitutions increased progressively with the sequential replacement of the XBB.1.9, BA.2.86, and XDV.1 lineages in Jining City during 2024–2025. Several substitutions identified in this study, including K356T, G446S, N450D, L452W, L455S, F456L, N460K, E554K, and A570V, have been reported to enhance immune escape capacity, whereas substitutions such as N440K, Q498R, and N501Y are known to increase binding affinity between SARS-CoV-2 and host receptors (Wang et al., 2023; Jian et al., 2023; Li and Zhang, 2022; Arbi et al., 2024). In addition, the T22N substitution identified in the spike protein of the XDV.1 lineage may introduce a novel N-linked glycosylation site, potentially hindering host antibody recognition and promoting immune escape (Li et al., 2025; Cao et al., 2025) The A435S substitution has been reported to enhance receptor binding affinity while reducing antibody-mediated neutralization (Li et al., 2025; Cao et al., 2025). Together, the T22N and A435S substitutions observed in the XDV.1 lineage may contribute to enhanced immune evasion and receptor binding, which could partially explain the widespread global transmission of the XDV.1 lineage since 2024. This indicates that with the continuous variation of the amino acids in the spike protein, the variations related to viral antigenic drift and those enhancing the binding affinity with host receptors are constantly accumulating, which may be the driving force for the continuous replacement of Omicron sublineages. Due to the continuous variation of the spike protein, the development of vaccines targeting structural proteins with high conservation, such as nucleocapsid protein and membrane protein, can be explored to reduce the risk of immune escape caused by mutations in a single spike protein target and improve the broad-spectrum protective effect of vaccines.

ORF8 is a multifunctional protein of SARS-CoV-2 that plays an important role in viral immune modulation. ORF8 can influence host immunity by mediating MHC-I degradation and inhibiting the type I interferon signaling pathway, and it can also trigger cytokine storm responses through activation of the IL-17 signaling pathway, thereby contributing to viral pathogenicity (Wang et al., 2025; Zhang et al., 2021; Li et al., 2020; Chen et al., 2022; Lin et al., 2021). However, ORF8 is prone to various forms of premature termination mutations, resulting in truncated ORF8 proteins, such as the ORF8 G8 stop and ORF8 Q18 stop mutations (Valcarcel et al., 2021; Vinjamuri et al., 2022). These premature stop mutations lead to the expression of only a short N-terminal segment of the ORF8 protein. Based on structural modeling predictions, the ORF8 G8 stop and Q18 stop mutations may result in the loss of its binding sites for IRF3 and IL-17RA, which may result in functional loss of ORF8, potentially leading to reduced viral pathogenicity, prolonged infection, or an increased rate of asymptomatic infection. Furthermore, ORF8 G8 stop is a characteristic feature of the XBB.1 lineage, emerging during its phase of diversification. This mutation reflects an evolutionary trade-off: while optimizing the Spike protein to enhance ACE2 binding affinity, the virus concurrently attenuates its pathogenicity, thereby facilitating efficient transmission of SARS-CoV-2 (Tamura et al., 2024). In addition, an A28254 deletion identified in some sequences caused a frameshift mutation in ORF8, resulting in the addition of a terminal peptide segment at the C-terminus. This alteration may affect the spatial conformation of the ORF8 protein and potentially confer novel functional properties, although further experimental studies are required to confirm these effects.

In summary, through whole-genome sequencing and bioinformatic analysis of 429 SARS-CoV-2 strains collected in Jining City during 2024–2025, this study expands the available genomic data and elucidates the sequential replacement pattern of SARS-CoV-2 lineages from XBB.1.9 to BA.2.86 and subsequently to XDV.1. Consequently, enhanced surveillance through robust SARS-CoV-2 testing and whole-genome sequencing is critical. These efforts must focus on tracking evolutionary trends in key genomic regions, specifically Spike protein mutations that confer immune escape or enhanced receptor binding, and ORF8 truncation variants. Monitoring these changes is vital for performing timely risk assessments and disseminating early warning alerts. Additionally, establishing information-sharing mechanisms with adjacent regions is necessary to maintain up-to-date situational awareness of circulating viral lineages.

## Data Availability

The original contributions presented in the study are included in the article/Supplementary material, further inquiries can be directed to the corresponding authors.

## Supplementary material

SUPPLEMENTARY FIGURE 1 Genomic characteristics of 429 SARS-CoV-2 sequences circulating in Jining City during 2024–2025. Sequence analysis was performed using Nextclade v3.18.1. The inner circle represents the major lineages, including XBB.1.9, BA.2.86, and XDV.1. The outer circle represents the corresponding sublineages of XBB.1.9, BA.2.86, and XDV.1.

SUPPLEMENTARY FIGURE 2 Genomic characteristics of 240 SARS-CoV-2 sequences circulating in Jining City in 2024. Sequence analysis was performed using Nextclade v3.18.1.

SUPPLEMENTARY FIGURE 3 Genomic characteristics of 189 SARS-CoV-2 sequences circulating in Jining City in 2025. Sequence analysis was performed using Nextclade v3.18.1.

SUPPLEMENTARY FIGURE 4 Spatial distribution of 429 SARS-CoV-2 genomes across 11 counties in Jining City during 2024–2025. The map depicts the geographic spread of the sequenced isolates and was generated using ArcGIS software.

SUPPLEMENTARY FIGURE 5 Amino acid variations in the spike protein of SARS-CoV-2 in Jining City during 2024–2025, compared to the Wuhan-Hu-1 reference sequence. **(a)** Amino acid variations in the spike protein of HK.3.2 sublineage sequences in Jining City during 2024–2025, compared to the Wuhan-Hu-1 reference sequence. **(b)** Amino acid variations in the spike protein of JN.1 sublineage sequences in Jining City during 2024–2025, compared to the Wuhan-Hu-1 reference sequence. **(c)** Amino acid variations in the spike protein of JN.1.4.5 sublineage sequences in Jining City during 2024–2025, compared to the Wuhan-Hu-1 reference sequence. **(d)** Amino acid variations in the spike protein of JN.1.16 sublineage sequences in Jining City during 2024–2025, compared to the Wuhan-Hu-1 reference sequence. **(e)** Amino acid variations in the spike protein of JN.1.67.1 sublineage sequences in Jining City during 2024–2025, compared to the Wuhan-Hu-1 reference sequence. **(f)** Amino acid variations in the spike protein of NB.1 sublineage sequences in Jining City during 2024–2025, compared to the Wuhan-Hu-1 reference sequence. **(g)** Amino acid variations in the spike protein of NB.1.8.1 sublineage sequences in Jining City during 2024–2025, compared to the Wuhan-Hu-1 reference sequence. **(h)** Amino acid variations in the spike protein of PQ.1 sublineage sequences in Jining City during 2024–2025, compared to the Wuhan-Hu-1 reference sequence. **(i)** Amino acid variations in the spike protein of PQ.2 sublineage sequences in Jining City during 2024–2025, compared to the Wuhan-Hu-1 reference sequence. **(j)** Amino acid variations in the spike protein of PQ.17 sublineage sequences in Jining City during 2024–2025, compared to the Wuhan-Hu-1 reference sequence. Sublineage specific mutation sites were queried using the China National Center for Bioinformation (CNCB) database (https://ngdc.cncb.ac.cn/ncov/knowledge/compare). Mutations specific to the sublineage are highlighted in red, whereas those unique to the Jining are marked in blue.
