# Supplementary material for: Comprehensive whole-genome characterization of SARS-CoV-2 strains in Jining China 2024–2025
Source: Front Microbiol. 2026 May 8;17:1798666. doi: 10.3389/fmicb.2026.1798666 (PMC13194450; doi:10.3389/fmicb.2026.1798666)
Supplement: Supplementary file 1 [file Table_1.docx]

Supplementary Table 1. Number of positive SARS-CoV-2 tests and number of comprehensive whole-Genome of SARS-CoV-2 obtained in Jining City during 2024–2025

| Date | Number of positive SARS-CoV-2 tests in Jining City | Number of comprehensive whole-Genome of SARS-CoV-2 obtained in Jining City | Proportion |
| --- | --- | --- | --- |
| January 2024 | 99 | 23 | 23.23% |
| February 2024 | 342 | 21 | 6.14% |
| March 2024 | 1049 | 44 | 4.19% |
| April 2024 | 320 | 35 | 10.94% |
| May 2024 | 77 | 11 | 14.29% |
| June 2024 | 39 | 11 | 28.21% |
| July 2024 | 108 | 10 | 9.26% |
| August 2024 | 1027 | 38 | 3.70% |
| September 2024 | 328 | 34 | 10.37% |
| October 2024 | 32 | 11 | 34.38% |
| November 2024 | 29 | 0 | 0.00% |
| December 2024 | 46 | 1 | 2.17% |
| January 2025 | 77 | 0 | 0.00% |
| February 2025 | 83 | 7 | 8.43% |
| March 2025 | 87 | 1 | 1.15% |
| April 2025 | 188 | 17 | 9.04% |
| May 2025 | 314 | 24 | 7.64% |
| June 2025 | 229 | 29 | 12.66% |
| July 2025 | 426 | 57 | 13.38% |
| August 2025 | 645 | 27 | 4.19% |
| September 2025 | 162 | 21 | 12.96% |
| October 2025 | 38 | 4 | 10.53% |
| November 2025 | 19 | 3 | 15.79% |
| December 2025 | 12 | 0 | 0.00% |
