# Supplementary material for: Comprehensive whole-genome characterization of SARS-CoV-2 strains in Jining China 2024–2025
Source: Front Microbiol. 2026 May 8;17:1798666. doi: 10.3389/fmicb.2026.1798666 (PMC13194450; doi:10.3389/fmicb.2026.1798666)
Supplement: Supplementary file 3 [file Table_3.docx]

Supplementary Table 3. Genetic characteristics of 429 SARS-CoV-2 strains circulating in Jining City, 2024–2025.

| Genetic characteristics of SARS-CoV-2 in 2024 | | | | | | Genetic characteristics of SARS-CoV-2 in 2025 | | | |
| --- | --- | --- | --- | --- | --- | --- | --- | --- | --- |
| Sublineage | Proportion | Sublineage | Proportion | Sublineage | Proportion | Sublineage | Proportion | Sublineage | Proportion |
| HK.3 | 0.42% | JN.1.9 | 0.42% | LZ.2.1.2 | 1.25% | NB.1 | 5.82% | PQ.2.4 | 1.06% |
| HK.3.13 | 1.67% | KP.1 | 0.42% | LZ.2.2 | 0.83% | NB.1.2.1 | 0.53% | PQ.2.5 | 0.53% |
| HK.3.2 | 12.50% | KP.1.1.3 | 2.08% | MU.3 | 0.42% | NB.1.3 | 0.53% | PQ.22 | 0.53% |
| JN.1 | 10.00% | KP.2 | 0.42% | MU.4 | 1.25% | NB.1.5.2 | 0.53% | PQ.26 | 0.53% |
| JN.1.1 | 0.42% | KP.2.2 | 0.83% | MZ.3 | 0.83% | NB.1.7 | 3.17% | PQ.3 | 0.53% |
| JN.1.1.9 | 0.42% | KP.2.25.1 | 1.67% | MZ.4 | 0.42% | NB.1.8 | 1.06% | PQ.31 | 1.59% |
| JN.1.16 | 17.92% | KP.2.3 | 0.83% | NB.1 | 1.25% | NB.1.8.1 | 25.40% | PQ.31.1 | 3.17% |
| JN.1.16.3 | 0.83% | KP.2.3.13 | 0.42% | NS.1.2 | 1.67% | PQ.1 | 7.94% | PQ.32 | 0.53% |
| JN.1.28 | 0.42% | KP.2.3.6 | 0.42% | XDD.1.1 | 0.42% | PQ.1.2 | 0.53% | PQ.4 | 2.12% |
| JN.1.30 | 0.83% | KP.3.2.6 | 2.50% | XDV.1 | 7.08% | PQ.10.1.4 | 0.53% | PQ.4.4 | 5.82% |
| JN.1.39 | 0.42% | KS.1.1.2 | 0.42% | XDV.1.1 | 2.08% | PQ.11 | 0.53% | PQ.4.5 | 0.53% |
| JN.1.4 | 3.33% | KW.1.1 | 0.42% | XDV.1.2.1 | 0.42% | PQ.17 | 10.58% | PQ.5 | 1.59% |
| JN.1.4.5 | 6.67% | LB.1 | 0.42% | XDV.1.3 | 0.42% | PQ.17.2 | 0.53% | PQ.7 | 3.70% |
| JN.1.5 | 0.42% | LB.1.2 | 1.67% | XDV.1.6 | 0.42% | PQ.17.6 | 1.59% | PQ.9 | 0.53% |
| JN.1.58 | 0.42% | LB.1.3 | 0.42% | XDV.1.7 | 1.67% | PQ.2 | 15.34% | PQ.9.1 | 0.53% |
| JN.1.67.1 | 5.83% | LB.1.3.2 | 0.42% | XDV.1.7.1 | 0.42% | PQ.2.1 | 0.53% | RH.1 | 0.53% |
| JN.1.69 | 0.42% | LB.1.5 | 0.42% | XDV.1.8 | 0.42% | PQ.2.1.10 | 0.53% | XFF.1 | 0.53% |
| JN.1.7 | 0.42% | LZ.2.1 | 1.25% |  |  |  |  |  |  |
