# Supplementary material for: Comprehensive whole-genome characterization of SARS-CoV-2 strains in Jining China 2024–2025
Source: Front Microbiol. 2026 May 8;17:1798666. doi: 10.3389/fmicb.2026.1798666 (PMC13194450; doi:10.3389/fmicb.2026.1798666)
Supplement: Supplementary file 4 [file Table_4.docx]

Supplementary Table 4. Sequence similarity analysis of predominant SARS-CoV-2 sublineage genomes compared with Wuhan-Hu-1

| Gene | HK.3.2 | | JN.1 | | JN.1.16 | | JN.1.4.5 | | JN.1.67.1 | | NB.1.8.1 | | PQ.2 | | PQ.17 | | PQ.1 | | NB.1 | |
| --- | --- | --- | --- | --- | --- | --- | --- | --- | --- | --- | --- | --- | --- | --- | --- | --- | --- | --- | --- | --- |
|  | Similarity ranges (%) | Average genetic distance | Similarity ranges (%) | Average genetic distance | Similarity ranges (%) | Average genetic distance | Similarity ranges (%) | Average genetic distance | Similarity ranges (%) | Average genetic distance | Similarity ranges (%) | Average genetic distance | Similarity ranges (%) | Average genetic distance | Similarity ranges (%) | Average genetic distance | Similarity ranges (%) | Average genetic distance | Similarity ranges (%) | Average genetic distance |
| ORF1ab | 99.76-99.82 | 0.002 | 99.82-99.87 | 0.002 | 99.80-99.84 | 0.002 | 99.79-99.83 | 0.002 | 99.82-99.85 | 0.002 | 99.80-99.85 | 0.002 | 99.78-99.81 | 0.002 | 99.79-99.83 | 0.002 | 99.73-99.82 | 0.002 | 99.79-99.82 | 0.002 |
| S | 98.69-98.81 | 0.013 | 98.60-98.65 | 0.014 | 98.36-98.41 | 0.016 | 98.40-98.49 | 0.015 | 98.71-98.76 | 0.013 | 98.69-98.79 | 0.012 | 98.52-98.58 | 0.014 | 98.57-98.70 | 0.013 | 98.72-98.80 | 0.012 | 99.00-99.06 | 0.010 |
| ORF3 | 99.28-99.52 | 0.005 | 99.64-99.56 | 0.003 | 99.64-99.76 | 0.003 | 99.64-99.76 | 0.003 | 99.64-99.76 | 0.003 | 99.51-99.75 | 0.003 | 99.39-99.51 | 0.005 | 99.76 | 0.002 | 99.52-99.76 | 0.003 | 99.52-99.76 | 0.004 |
| E | 98.68-99.12 | 0.009 | 99.56 | 0.004 | 99.56-99.56 | 0.004 | 99.56 | 0.004 | 99.56 | 0.004 | 99.12-99.56 | 0.004 | 99.56 | 0.004 | 100 | 0 | 99.56 | 0.004 | 99.56 | 0.004 |
| M | 99.40-99.55 | 0.005 | 99.40 | 0.006 | 98.80-98.95 | 0.011 | 98.95 | 0.010 | 99.40 | 0.006 | 99.10-99.25 | 0.008 | 98.95-99.10 | 0.009 | 99.25-99.40 | 0.006 | 98.95-99.10 | 0.009 | 99.40-99.55 | 0.005 |
| ORF6 | 97.31-97.85 | 0.022 | 97.85 | 0.022 | 97.85 | 0.022 | 97.85 | 0.022 | 97.85 | 0.022 | 97.84-98.38 | 0.016 | 98.38 | 0.016 | 97.31-97.85 | 0.022 | 98.38 | 0.016 | 97.85 | 0.022 |
| ORF7a | 99.45-99.73 | 0.003 | 99.73-100 | 0.0004 | 99.73-100 | 0.0002 | 99.73-100 | 0.0003 | 100 | 0 | 99.72-100 | 0.0002 | 99.73-100 | 0.0002 | 99.73-100 | 0.0001 | 99.73-100 | 0.0002 | 100 | 0 |
| ORF8 | 98.63-99.73 | 0.004 | 99.73-100 | 0.0002 | 99.73-100 | 0.0001 | 99.44-99.72 | 0.003 | 99.45-100 | 0.001 | 99.45-100 | 0.0004 | 99.73-100 | 0.0001 | 99.73-100 | 0.0001 | 99.45-100 | 0.001 | 99.73-100 | 0.001 |
| N | 99.44-99.552 | 0.005 | 99.36-99.52 | 0.005 | 99.36-99.52 | 0.005 | 99.36-99.52 | 0.005 | 99.44-99.52 | 0.005 | 99.36-99.44 | 0.006 | 99.44-99.52 | 0.005 | 99.36-99.44 | 0.006 | 99.28-99.36 | 0.007 | 99.36-99.44 | 0.006 |
| ORF10 | 98.97-100 | 0.010 | 100 | 0 | 100 | 0 | 98.97-100 | 0.001 | 100 | 0 | 98.97-100 | 0.001 | 100 | 0 | 100 | 0 | 98.97-100 | 0.001 | 98.97-100 | 0.001 |
