# Supplementary figures and images for: Comprehensive whole-genome characterization of SARS-CoV-2 strains in Jining China 2024–2025
Source: Front Microbiol. 2026 May 8;17:1798666. doi: 10.3389/fmicb.2026.1798666 (PMC13194450; doi:10.3389/fmicb.2026.1798666)

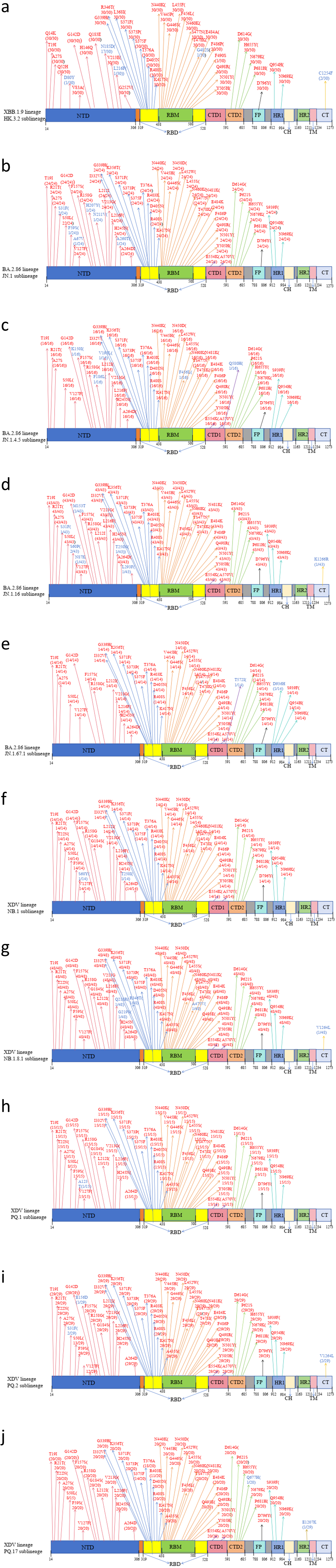

Supplement: SUPPLEMENTARY FIGURE 5 — Amino acid variations in the spike protein of SARS-CoV-2 in Jining City during 2024–2025, compared to the Wuhan-Hu-1 reference sequence. (a) Amino acid variations in the spike protein of HK.3.2 sublineage sequences in Jining City during 2024–2025, compared to the Wuhan-Hu-1 reference sequence. (b) Amino acid variations in the spike protein of JN.1 sublineage sequences in Jining City during 2024–2025, compared to the Wuhan-Hu-1 reference sequence. (c) Amino acid variations in the spike protein of JN.1.4.5 sublineage sequences in Jining City during 2024–2025, compared to the Wuhan-Hu-1 reference sequence. (d) Amino acid variations in the spike protein of JN.1.16 sublineage sequences in Jining City during 2024–2025, compared to the Wuhan-Hu-1 reference sequence. (e) Amino acid variations in the spike protein of JN.1.67.1 sublineage sequences in Jining City during 2024–2025, compared to the Wuhan-Hu-1 reference sequence. (f) Amino acid variations in the spike protein of NB.1 sublineage sequences in Jining City during 2024–2025, compared to the Wuhan-Hu-1 reference sequence. (g) Amino acid variations in the spike protein of NB.1.8.1 sublineage sequences in Jining City during 2024–2025, compared to the Wuhan-Hu-1 reference sequence. (h) Amino acid variations in the spike protein of PQ.1 sublineage sequences in Jining City during 2024–2025, compared to the Wuhan-Hu-1 reference sequence. (i) Amino acid variations in the spike protein of PQ.2 sublineage sequences in Jining City during 2024–2025, compared to the Wuhan-Hu-1 reference sequence. (j) Amino acid variations in the spike protein of PQ.17 sublineage sequences in Jining City during 2024–2025, compared to the Wuhan-Hu-1 reference sequence. Sublineage specific mutation sites were queried using the China National Center for Bioinformation (CNCB) database (https://ngdc.cncb.ac.cn/ncov/knowledge/compare). Mutations specific to the sublineage are highlighted in red, whereas those unique to the Jining are marked in blue. [file Image_5.TIF]
